# Recurrence of Cervical Squamous Cell Carcinoma After More Than 4 Years in the Lung: A Rare Case Report

**DOI:** 10.7759/cureus.41599

**Published:** 2023-07-09

**Authors:** Asmae Boudouh, Hajar Charii, Amine Hayoune, Mohammed Aharmim, Jamal Eddine Bourkadi

**Affiliations:** 1 Department of Respiratory Diseases, Mohammed VI University Hospital, Oujda, MAR; 2 Department of Respiratory Diseases, Moulay Youssef Hospital, Rabat, MAR

**Keywords:** cancer, gynecologic, metastatic, lung, cervical squamous cell carcinoma, recurrence, case report

## Abstract

Cervical squamous cell carcinoma (CSCC) is a common gynecological malignancy, typically affecting women of reproductive age. Although the occurrence of late metastatic recurrence in the lung is relatively rare, we present the case of a 52-year-old woman, previously diagnosed and treated for CSCC. After 4 years of disease-free intervals, she presented with respiratory symptoms, including cough, dyspnea, and hemoptysis, with marked asthenia. A computed tomography (CT) scan of the chest revealed a lung mass. Histopathological examination of the lung biopsy confirmed the recurrence of CSCC, specifically in the lung. Immunohistochemistry further supported the origin of the tumor as cervical.

The management of such cases necessitates a multidisciplinary approach, considering treatment options such as surgery and chemoradiation. Long-term follow-up and surveillance are vital for the early detection of late recurrences, as they can present several years after the initial diagnosis.

This case report highlights the importance of recognizing and appropriately managing cases of late metastatic recurrence of CSCC in the lung. Further studies are needed to deepen our understanding of the underlying mechanisms, refine diagnostic approaches, and optimize treatment strategies for such rare occurrences.

## Introduction

Cervical squamous cell carcinoma (CSCC) is one of the most common gynecological malignancies, primarily affecting women of reproductive age. However, metastatic spread to distant organs is relatively rare, particularly in the late stages of the disease [1].

Late metastatic CSCC refers to the dissemination of cancer cells beyond the primary site after a significant period following the initial diagnosis and treatment [1]. Metastasis to distant organs, including the lungs, is infrequent in cervical cancer cases [1]. The occurrence of bronchial infiltration further accentuates the rarity of the presentation [2]. Understanding and recognizing such exceptional cases is crucial for accurate diagnosis, appropriate treatment strategies, and improved patient outcomes.

## Case presentation

 A 52-year-old woman, postmenopausal for nine years, was treated in 2019 for CSCC revealed by postmenopausal bleeding, with the presence of a budding lesion at the expense of the uterine cervix on gynecological examination. Hysteroscopy revealed a moderately differentiated squamous cell carcinoma infiltrating the uterine cervix. The patient underwent a hysterectomy and bilateral salpingo-oophorectomy, followed by adjuvant chemoradiation.

**Figure 1:**
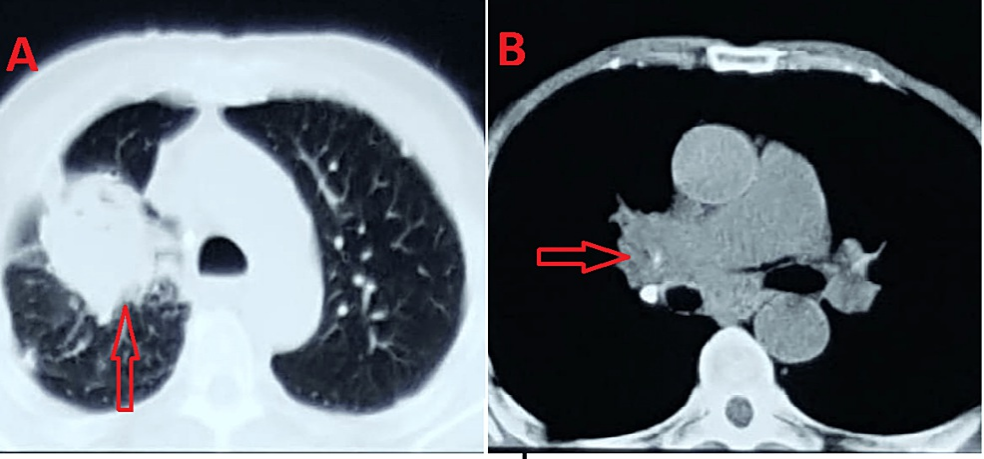
CT scan with red arrows pointing to lung mass. A: axial section with parenchymal window. B: axial section with mediastinal window.

After four years of regular follow-up, the patient presented with respiratory symptoms consisting of dyspnea, cough, and hemoptysis, with marked asthenia. The chest computed tomography (CT) scan showed a lung mass, and a collapse of the ventral segment of the right upper lobe and the middle lobe, with mediastinal lymphadenopathy (Figure 1).

**Figure 2:**
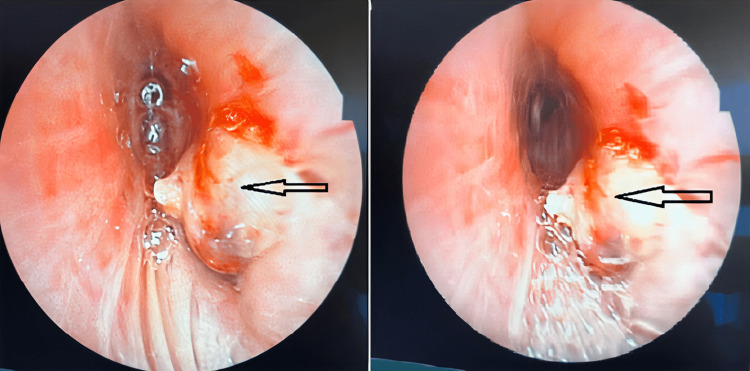
Bronchoscopy showed a tumoral growth at the end of the trachea (black arrow).

Bronchoscopy revealed the presence of a necrotic bud completely obstructing the middle lobar (Figure 2).

**Figure 3:**
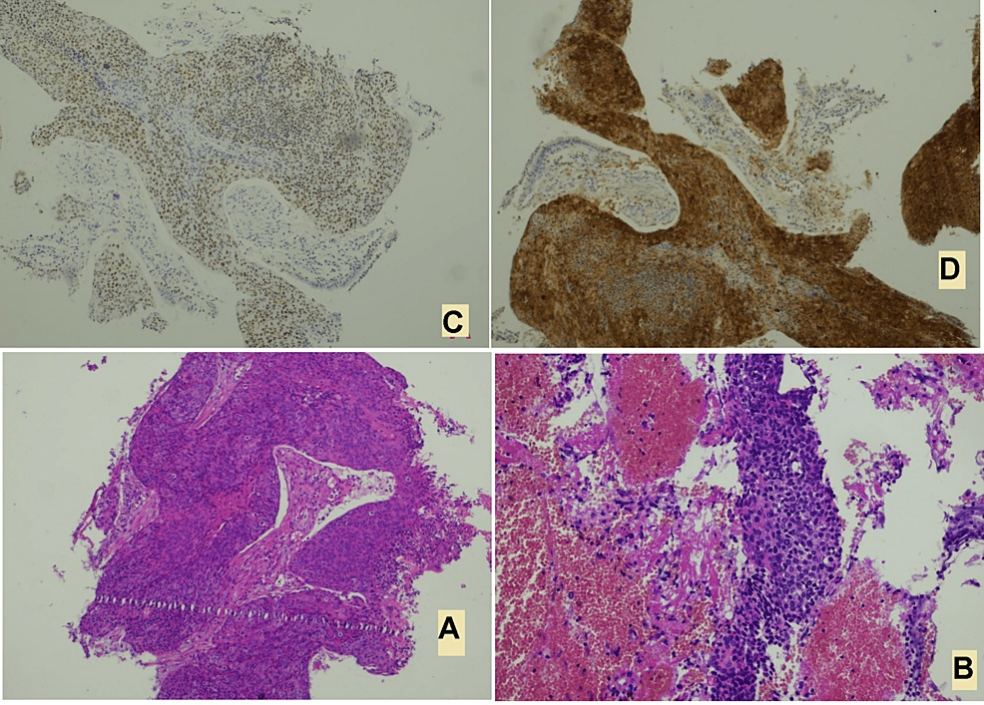
Histology showing squamous cell carcinoma of the cervix (A,B) and immunohistochemical stain for surrogate high-risk HPV markers: p16 and p40 (D,C).

The histologic examination of the samples revealed invasion of the bronchial mucosa by moderately differentiated squamous cell carcinoma, whose immunohistochemical staining showed strong positivity for P16 and P40, which aligns with a profile consistent with a cervical origin (Figure 3).

Brain and abdominopelvic scans were performed as part of the extension workup, and they were normal. The patient is referred to medical oncology for therapeutic management, She shows a positive clinical response after starting chemotherapy consisting of four cycles of cisplatin and paclitaxel. The evolution of the patient's condition is ongoing.

## Discussion

The recurrence of CSCC in the lung is a rare occurrence, accounting for approximately 0.7-2.2% of all cases of cervical cancer recurrence [1]. In this case report, we presented a unique case of isolated lung recurrence of CSCC, highlighting the importance of recognizing and managing such cases appropriately.

The development of distant metastases in CSCC usually involves regional lymph nodes, the liver, and the lungs [1]. However, isolated lung recurrence, without the involvement of other sites, is relatively uncommon. The exact mechanisms underlying this phenomenon are not well understood, but it is believed to involve a combination of factors, including tumor biology, lymphatic and hematogenous spread, and the presence of favorable microenvironments in the lung for tumor growth and colonization.

The clinical presentation of isolated lung recurrence can vary and may include respiratory symptoms such as cough, dyspnea, and hemoptysis, as seen in our patient. Imaging studies play a crucial role in the evaluation of suspected lung recurrence, and a high index of suspicion is necessary, especially in patients with a history of CSCC [3]. In our case, it was identified on imaging, prompting further investigations.

Histopathological examination of the lung biopsy confirmed the presence of metastatic CSCC, providing conclusive evidence of recurrence. Accurate histological confirmation is essential to differentiate recurrence from primary lung malignancies or other lung metastases originating from different primary tumors. The main histological type is squamous cell carcinoma of the cervix, adenocarcinoma, and adenosquamous carcinoma, with some other less common histological test types of neuroendocrine tumors sarcoma [4]. Immunohistochemistry can be employed to further support the diagnosis and identify the tumor's origin.

The management of isolated lung recurrence in CSCC requires a multidisciplinary approach [5,2]. Treatment options may include surgery, radiation therapy, or systemic therapy depending on factors such as the extent of the recurrence, the patient's overall health, and the presence of other sites of metastasis [5,2]. The goal is to achieve local control of the disease while minimizing treatment-related toxicity and preserving the quality of life.

Long-term follow-up is crucial in patients with CSCC, even after successful initial treatment, as late recurrences can occur. The recurrence pattern may vary among individuals, emphasizing the need for continued surveillance and vigilance. Monitoring for signs and symptoms of recurrence, regular imaging studies, and appropriate diagnostic evaluations are essential components of post-treatment care.

## Conclusions

The recurrence of CSCC in the lung is a rare manifestation, accounting for a small percentage of cervical cancer recurrences. Our case report highlights the importance of considering this possibility in patients presenting with respiratory symptoms and a history of CSCC. Further studies and reports are needed to enhance our understanding of the underlying mechanisms, optimal diagnostic approaches, and effective treatment strategies for such cases.
